# A Comparison of the Compositional, Microstructural, and Mechanical Characteristics of Ni-Free and Conventional Stainless Steel Orthodontic Wires

**DOI:** 10.3390/ma12203424

**Published:** 2019-10-19

**Authors:** Daniela Brüngger, Theodoros Koutsoukis, Youssef S. Al Jabbari, Monika Hersberger-Zurfluh, Spiros Zinelis, Theodore Eliades

**Affiliations:** 1 Clinic of Orthodontics and Pediatric Dentistry, Center of Dental Medicine, Faculty of Medicine, University of Zurich, 8032 Zurich, Switzerland; dani.bruengger@bluewin.ch; 2 Dental Biomaterials Research and Development Chair, College of Dentistry, King Saud University, Riyadh 11545, Saudi Arabia; theodoroskoutsoukis@gmail.com (T.K.); szinelis@dent.uoa.gr (S.Z.); 3 Department of Prosthetic Dental Sciences, College of Dentistry, King Saud University, Riyadh 11545, Saudi Arabia; 4 Department of Biomaterials, School of Dentistry, National and Kapodistrian University of Athens, 11527 Athens, Greece; monika.hersberger-zurfluh@zzm.uzh.ch

**Keywords:** orthodontic wires, Ni supersensitive, microstructure, SEM, EDX, XRD, IIT, SS

## Abstract

Ni-free orthodontic wires were introduced to mitigate concerns associated with the use of Ni-containing alloys in orthodontics. However, limited information is available on their properties and therefore, the aim of this study was to characterize the elemental composition, the microstructure, and the mechanical properties of Ni-free orthodontic wires and compare them with their stainless steel (SS) counterparts. Four Ni-free and four conventional SS wires were included in this study. All the wires were initially imaged with a Scanning Electron Microscopy (SEM) and their elemental compositions were determined by X-ray Energy Dispersive Spectroscopy (EDX). Then, their microstructure was assessed by X-ray Diffraction (XRD) and the indentation modulus, elastic index, Martens Hardness and Vickers Hardness by Instrumented Indentation Testing (IIT). All the wires demonstrated surface cracks and pores oriented parallel to their long axis. The elemental composition of Ni-free alloys showed an increased Mn and Cr content while both SS and Ni-free wires shared the same dominant austenite structure. In conclusion, despite the differences in elemental composition, Ni-free wires demonstrated a similar microstructure and comparable mechanical properties with their conventional SS counterparts and thus may be considered as a promising alternative for patients with Ni supersensitivity.

## 1. Introduction

Although orthodontic wires and brackets made of Ni-containing stainless steel (SS) alloys are extensively used in modern orthodontic therapy, the possible adverse biological consequences of Ni are a well-documented and highly investigated topic in dental literature [1,2,3]. The majority of brackets and wires are made of austenitic type AISI (American Iron and Stainless Steel Institute) 304 SS alloy [4,5] although AISI 303 AISI 316 have also been identified in some commercial products [4]. All the above mentioned alloys have a nominal composition with Ni content above 8 wt% [4,5] and patients previously sensitized to Ni are more vulnerable to allergic response to Ni-containing alloys [6].

In an effort to prevent the adverse effects of Ni in allergic patients, new orthodontic wires made of Ni-free SS alloys were introduced [7,8,9,10]. However, the substitution of Ni by other elements may provoke alterations in the microstructural, mechanical and corrosion resistance of SS alloys, as the presence of Ni has a notable effect on these aforementioned characteristics.

SS alloys are classified based on their crystal structure in ferrite, austenite and martensite. However, the austenite structure is preferable as it combines greater ductility and weldability and a greater degree of cold working and increased corrosion resistance [10,11]. From a metallurgical standpoint, Ni stabilizes the austenite structure at lower temperatures [3,12], providing ductility and other desirable mechanical properties and an increased corrosion resistance [10]. Therefore, the change in the elemental composition of SS alloys resulting from the substitution of Ni by other elements may have an effect on microstructure, which may impact the stiffness, strength, resilience, formability, weldability, corrosion resistance, and other clinically important properties [12].

Therefore, the aim of this study was to evaluate the aforementioned properties of orthodontic wires made of Ni-free alloys and to compare them with their SS counterparts. The null hypothesis of this study was that there are no differences in the microstructure and mechanical properties of SS and Ni-free orthodontic wires.

## 2. Materials and Methods

### 2.1. Materials Tested

The nominal elemental compositions (wt%) of the materials used in this study are presented in Table 1 per manufacturers’ reported data. The elemental composition for the specimen entitled “Acme SS” was not received, but according to the manufacturing company, this product is described as the conventional 304 stainless steel grade (S30400 according to UNS designation) produced by the Vacuum Air Remelted (VAR) method. This composition is provided in Table 1 as taken from the ASM Metals Handbook [13]. The elemental composition of “LeoWire” is not provided. The codes stand for the first two letters of the brand name, and NF and SS stand for Nickel-free or SS alloys, respectively.

### 2.2. SEM/EDX Analysis

For the characterization of surface morphology and elemental composition, all the materials were analyzed by employing Scanning Electron Microscopy (SEM) (JSM 6610LV, Jeol Ltd., Tokyo, Japan) and X-ray Electron Dispersive Spectroscopy (EDX) (Oxford Instruments, Abingdon, UK). One segment from each wire (approximately 5 mm in length) was cleaned in an ultrasonic bath for 10 min and was put into the SEM chamber for analysis. Imaging and EDX analyses were performed at the surface of each wire using backscattered electrons (BE) emission at 25 kV voltage, 78 μA beam current, and 3000× nominal magnifications. The experimental conditions of the EDX analysis included an area analysis (collecting window 120 × 90 μm) from the surface of the specimens at 25 kV voltage with an acquisition time of 200 s, at a working distance of 11 mm. Three spectra were acquired from each material, the quantitative results were averaged, and the standard deviation was calculated.

### 2.3. XRD Analysis

Ten segments (approximately 5 mm in length) from each wire were mounted parallel to each other and an X-ray Diffraction (XRD) analysis was employed for the characterization of the constituent phases. One spectrum was collected from each orthodontic wire by employing an XRD machine (D8 Advance, Bruker, Billerica, MA, USA) with the experimental conditions shown in Table 2.

### 2.4. Instrumented Indentation Testing (IIT)

Ten segments (about 10 mm in length) were cut from each wire by employing orthodontic pliers and then were embedded longitudinally in epoxy resin (Epofix, Struers, Belarup, Denmark). Then, the specimens were metallographically ground with SiC water coolant paper from 400 up to 2000 grit and polished up to 1 μm alumina slurry in a grinding/polishing machine (Dap V, Struers). Finally, they were cleaned in an ultrasonic water bath for 10 min.

A universal hardness testing machine ZHU0.2/Z2.5 (Zwick Roell, Ulm, Germany) was used for IIT measurements. Force indentation depth curves were recorded by applying 9.8 N with 2 s dwell time by a Vickers indenter. Five readings were taken from the surface of each segment and the mean value was used as representative of the segment itself. (*n* = 10 per product). All force-indentation depth curves were recorded and Martens Hardness (HM), indentation modulus (E_IT_), and elastic index (η_IT_), were estimated according to the ISO 14577-1 specification [14]. The analytical formulas were given by the ISO 14577-1 and can also be found in recent publications [7,15]. Then, Vickers hardness was determined by measuring the diagonal length of indentations.

### 2.5. Statistical Analysis

The results of E_IT_ and η_IT_, HM, and HV were statistically analyzed by 1-way ANOVA, employing “material” as the discriminating variable. Significant differences among groups were determined by a post hoc Student–Newman–Keuls (SNK) multiple comparison analysis at a = 0.05.

## 3. Results

### 3.1. SEM/EDX Results

**Figure 1:**
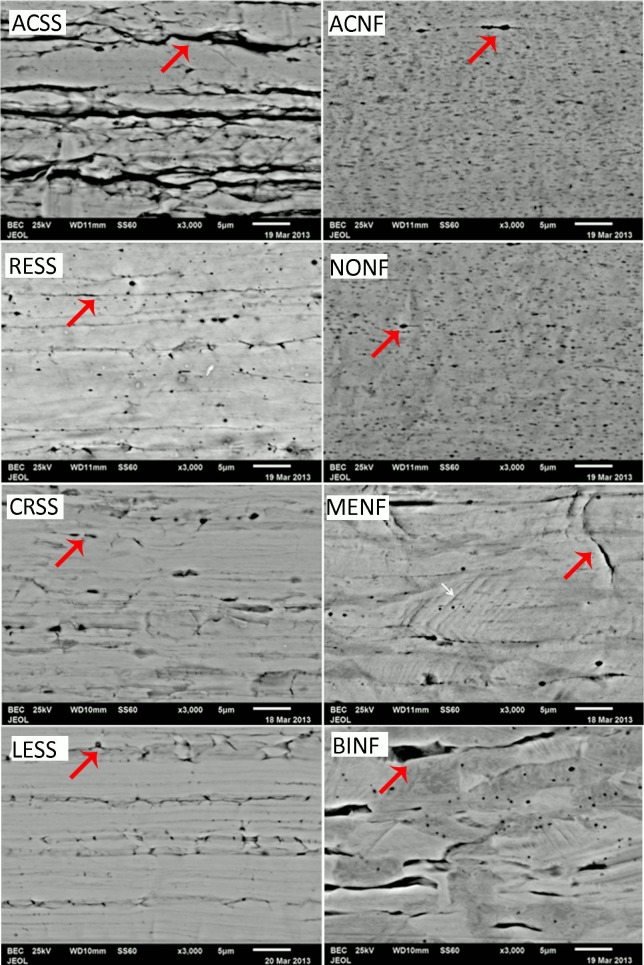
Representative BE images from the surfaces of all wires tested. The left column shows SS alloys and the right column Ni-free ones. The nominal magnification was 3000×. All images have the same orientation with the long axis of wires parallel to the horizontal axis of the images. The red arrows point to the cracks and pores while the white arrow (MENF) indicates the characteristic gliding of the planes that form a 45° angle with the long axis.

Figure 1 illustrates representative BE images of the surface of all wires tested. The long axis of all the wires examined is parallel to the horizontal axis of the images. Although different in size, all the materials demonstrated the presence of cracks and pores (red arrows) parallel to the long axis of the wires. ACSS and BINF illustrated the longer cracks while ACNF and NONF showed the shortest ones. The MENF also presented the characteristic gliding of the planes that form a 45° angle with the long axis of the wires (pointed by the white arrows in Figure 1).

**Figure 2:**
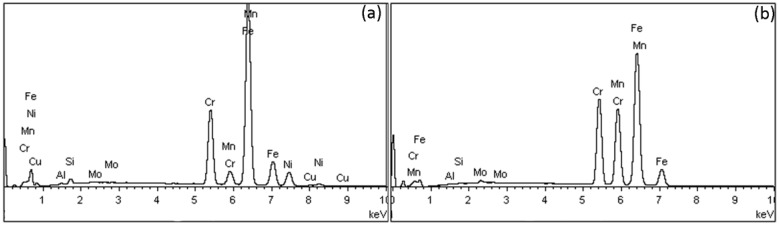
Representative EDS spectra from stainless steel (SS) alloys (**a**) and Ni-free alloys (**b**).

Figure 2 depicts EDX spectra from SS (a) and Ni-free alloys (b). The former present the characteristic peak of Ni and Cu, which vanished in the spectra of Ni-free alloys (except for MENF and BINF where a small amount of Ni was identified according to their nominal compositions (Table 1)).

Table 3 shows the elemental composition after EDX analysis (wt%) of all wires tested. All Ni-free alloys have Ni composition of less than 0.2 wt%, are free of Cu, and have an increased Mn and Cr content.

### 3.2. XRD Results

**Figure 3:**
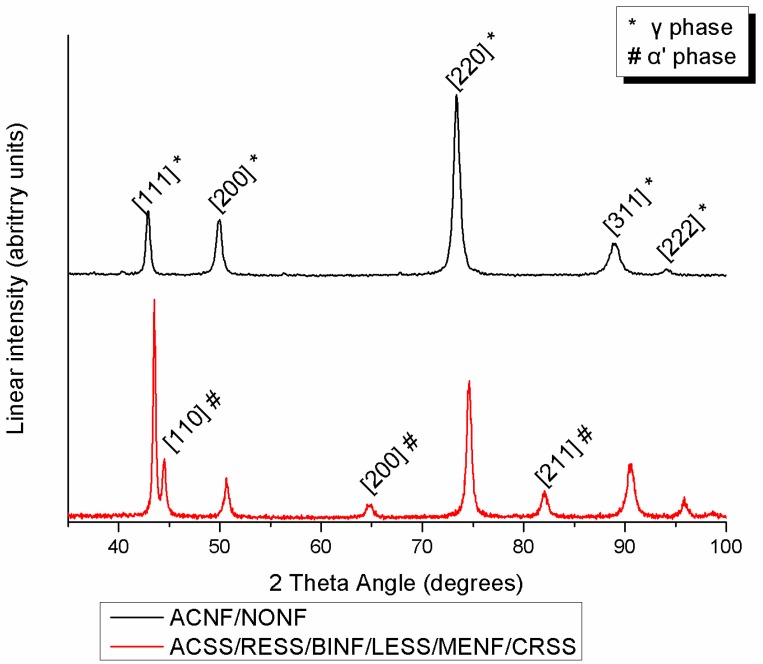
Indexed XRD spectra from all materials tested.

Figure 3 presents the indexed XRD diagrams of all the tested specimens. ACNF and NONF are comprised of austenite (γ phase) while all the rest also show the presence of martensite (α′ phase).

### 3.3. IIT Results

**Figure 4:**
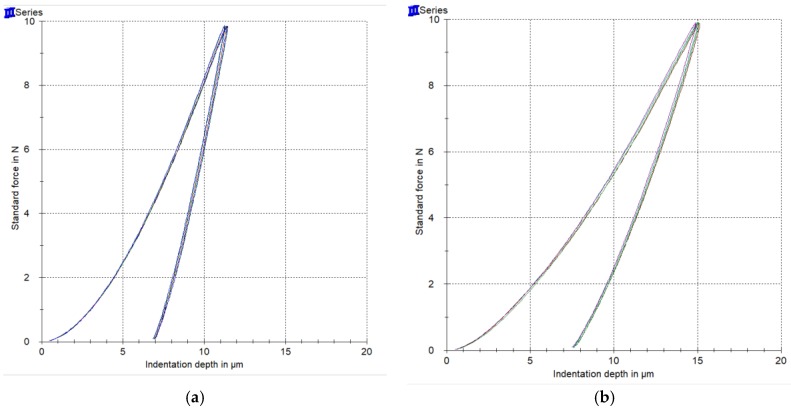
Representative force-indentation depth curves from a harder (**a**) and a softer (**b**) wire.

**Figure 5:**
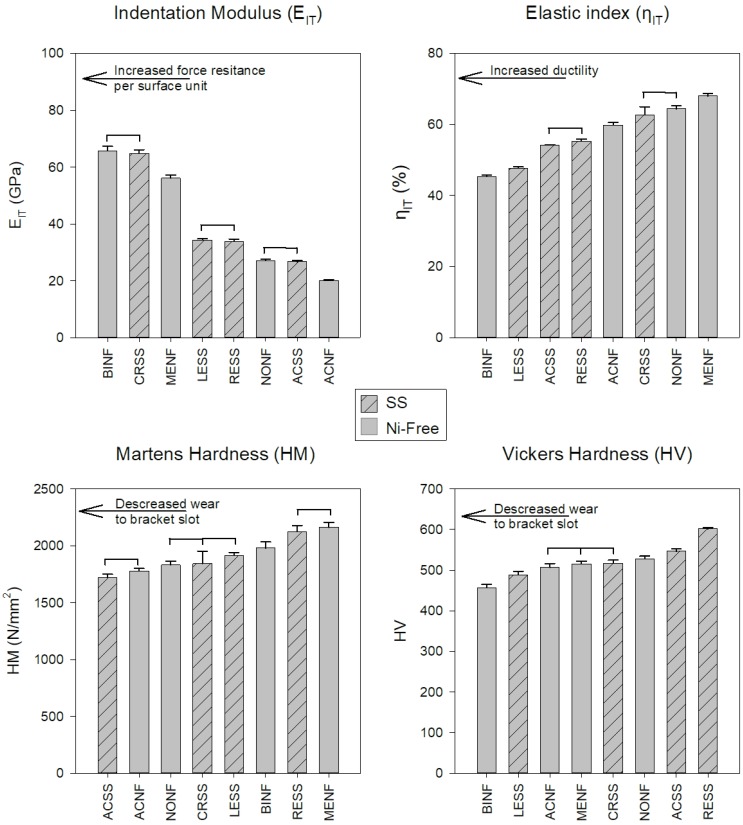
Mean values and standard deviations of all the materials tested. The bars with the internal pattern correspond to the SS alloys while the rest to the Ni-free ones. The horizontal lines connect the materials without statistically significant differences (*p* > 0.05). All the properties were sorted in ascending or descending order, starting from the left with the material with the best value according to the clinical implications for each property.

Representative force-indentation depth curves for a harder (a) and a softer (b) orthodontic wire are presented in Figure 4, as deeper indentation depth indicates a softer material. The results of E_IT_, η_IT_, HM, and HV are presented in Figure 5 along with the statistical outcome. The values are sorted in ascending or descending order, starting from the left with the wire with the best value according to the clinical implication for each property.

## 4. Discussion

According to the results of this study, the null hypothesis must be rejected as significant differences were found in the mechanical properties of the wires tested.

All the wires showed the presence of surface cracks and pores, a finding which is in accordance with previous reports [16,17,18]. It is well known that orthodontic wires undergo extensive cold working and the orientation of cracks and pores parallel to the long axis of wires should be appended to this manufacturing process [19]. Based on the results of the elemental composition after the EDX analysis (Table 2), Ni was not identified for two Ni-free alloys (ACNF, NONF) while Ni traces (<0.2 wt%) were identified for MENF and BINF. For all Ni-free alloys, Ni was replaced mainly by an increased Mn and Cr content, a finding which is in accordance with the nominal composition of these alloys as provided by the manufacturers (Table 1). In a few reports, Ni-free alloys are also called Manganese steels due to the increased presence of Mn [8]. Cu was only identified for SS alloys and although it was not mentioned in their nominal composition, Al was found to be present in all the wires tested, a finding which may be appended to surface grinding or polishing of these wires during the manufacturing process.

According to the elemental compositions derived, the increased amounts of austenite stabilizing elements such as Ni (more than approximately 8 wt%) or Mn prove that all the SS studied belong to the family of austenitic SS. This explains why in all the groups, the face centered cubic (fcc) austenitic phase (γ phase) was detected after the XRD analysis as the major phase of the microstructure. Moreover, the body centered tetragonal martensite (α′ phase) was detected in all spectra except ACNF and NONF. Martensite was formed by the transformation of austenite during cold deformation via TRIP (transformation-induced plasticity), a mechanism which explains the phenomenon of strain-induced martensite that takes place during the cold drawing of orthodontic wires [11,20,21]. The presence of both austenite and martensite phases is in full accordance with previous findings for conventional SS alloys [11]. In the Ni-free group, the peaks of the γ phase shifted to the right due to the smaller atomic radius of Mn compared to Ni.

SS and Ni-free alloys cannot be categorized in a hierarchal way as better or worse as they present mixed values, implying that the mechanical properties are not controlled solely by the differences in elemental composition. Therefore, Ni-free alloys cannot be classified as superior or inferior compared to their SS counterparts from the standpoint of mechanical properties. The E_IT_ values of all the wires tested were found to be much lower than the nominal values of the SS orthodontic wires ranging from 168 to 226 GPa [18,22]. This difference may be assigned to the fact that residual stresses induced during cold drawing strongly affect the unloading curve and thus the proper estimation of the selected value by IIT, a known complication of this methodology [23]. Therefore, the results of E_IT_ cannot be considered as conclusive (Figure 1) and an alternative methodology (i.e., tensile testing) must be employed for the proper determination of the modulus of elasticity.

Elastic index (Figure 5) is indicative of the ductility of the materials where the lower elastic indexes indicate more ductile alloys. In general, ductility is beneficial as it facilitates formability and hinders brittle fracture; from this standpoint, lower elastic indices are desirable. The elastic indices of this study are comparable with the results of a previous study for Ni-free and SS orthodontic wires [7]. However, the elastic index values were found to be higher than the expected values for ductile materials (<30%) [24] and this is in accordance with the limited strain at fracture (<3.7%) after tensile testing of orthodontic wires [25].

To the best of our knowledge, there is only one study with HM data for SS and Ni-free alloys in dental literature HM data [7] and thus, for comparison purposes with previous data, HV was also recorded in this study. Both HM and HV values are in accordance with previous data (1831~2656 HM) [7]) and (484~600 HV) [7,26,27,28], respectively. Interestingly, the two methods did not provide the same classifications of tested wires (Figure 5). This discrepancy is associated with the inherent limitation of Vickers testing, including the resolution of the optical system, the user’s perception, and most importantly, the rebound of material around indentation after load removal [29]. From a clinical standpoint, hardness is strongly associated with the wear of the slot walls where the wire comes into contact with the bracket slot surfaces. A recent study reported that wires with higher hardness demonstrated increased wear resistance against metallic brackets made of AISI 316 SS or Ti-6Al-4V alloy [28]. In order to minimize the wear at the wire slot interface of both wires and brackets, materials with matching hardness values should be used.

## 5. Conclusions

Both SS and Ni-free wires share the same dominant austenite structure.

Despite the significant differences among the different wires, SS and Ni-free alloys cannot be characterized as inferior or superior based on the mechanical properties tested.

## Figures and Tables

**Table 1:** Brand name, code and nominal elemental composition (wt%) of the tested materials.

Material/Code	Fe	Cr	Ni	Mo	Mn	Si	P	S	Other
Acme SS ^1^/ACSS	Bal	18.0–20.0	8.0–10.5		2.0	1.0	0.045	0.03	C ≤ 0.08
Acme Ni-Free ^1^/ACNF	Bal	21.0	≤0.1	0.7	23.0	≤0.75	≤0.03	≤0.01	Cu ≤ 0.25 N: 0.97 C ≤ 0.08
Remanium ^2^/RESS	Bal	18.0–20.0	8.0–10.5		≤2.0	≤1.0	≤0.045	≤0.03	C ≤ 0.08
Nominium ^2^/NONF	Bal	16.0–20.0	≤0.2	1.8–2.5	16.0–20.0	≤1.0	≤0.05	≤0.05	V ≤ 0.2 N: 0.7–1.0 C ≤ 0.1
Chromium ^4^/CRSS	Bal	18.0–20.0	6.0–9.0	≤0.8	≤2.0	≤1.5	≤0.045	≤0.03	C ≤ 0.12
Menzanium ^4^/MENF	Bal	16.0–20.0	≤0.2	1.8–2.5	16.0–20.0	≤1.0	≤0.005	≤0.05	V ≤ 0.2 N: 0.7–1.0 C ≤ 0.1
LeoWire ^3^/LENF	Not available								
BioSteel ^3^/BINF	Bal	18.0	0.2	2.0	18.0				N: 1.0

^1^ Acme Monaco, New Britain, CT, USA. ^2^ Dentaurum, Inspringen, Germany. ^3^ Scheu, Iserlohn, Germany. ^4^ Leone, Firenze, Italy.

**Table 2:** Experimental conditions for the XRD analysis of all the groups.

Radiation	CuKa
Voltage	40 V
Current	30 mA
Scan range (2θ angle)	35°–100°
Scan speed	0.02°/s
Scan step	0.02°
Preset time	1 s
Duration per run	72 min

**Table 3:** Mean values and standard deviations of the elemental compositions of all materials tested after EDS analysis (*n* = 3).

Material	Fe	Cr	Ni	Mo	Mn	Si	Al	Cu
ACSS	70.8 ± 0.2	18.9 ± 0.1	7.9 ± 0.1	0.4 ± 0.1	1.3	0.3	0.3	0.3
ACNF	53.0 ± 0.4	22.2 ± 0.3	BDL	0.9 ± 0.1	23.4	0.2	0.2 ± 0.1	BDL
RESS	70.8 ± 0.2	18.4 ± 0.8	8.1 ± 0.1	0.4 ± 0.1	1.2 ± 0.1	0.6 ± 0.5	0.3	0.3 ± 0.1
NONF	53.3 ± 0.2	22.0	BDL	0.8	23.3 ± 0.2	0.3	0.3	BDL
CRSS	70.3 ± 0.1	18.0 ± 0.1	8.0	0.5 ± 0.1	1.5 ± 0.1	1.0 ± 0.1	0.3	0.4
MENF	63.1 ± 0.3	19.4 ± 0.1	0.1	2.4 ± 0.1	13.6 ± 0.1	0.7	0.3	BDL
LESS	71.4 ± 0.3	17.5	8.4	0.7 ± 0.2	1.3	0.4	0.3	0.2
BINF	62.4 ± 0.4	20.2 ± 0.3	0.2	2.8	13.2 ± 0.9	1.1 ± 0.2	0.3	BDL

BDL: Below Detection Limit.
